# Hereditary oral squamous cell carcinoma associated with *CDKN2A* germline mutation: a case report

**DOI:** 10.1186/s40463-022-00556-y

**Published:** 2022-02-05

**Authors:** Ah-Reum Jeong, Kimberly Forbes, Ryan K. Orosco, Ezra E. W. Cohen

**Affiliations:** 1 Division of Hematology and Oncology, Department of Medicine, University of California San Diego, 3855 Health Sciences Drive, La Jolla, CA 92093-0960 USA; 2 Division of Otolaryngology, Department of Surgery, University of California San Diego, La Jolla, CA 92093 USA; 3 Moores Cancer Center, University of California San Diego, La Jolla, CA 92093 USA

**Keywords:** CDKN2A germline mutation, Familial atypical multiple moles melanoma, Head and neck squamous cell cancer, Oral squamous cell cancer, Case report

## Abstract

**Background:**

Germline *CDKN2A* mutations are a well-known cause of familial atypical multiple mole melanoma (OMIM #155601) and melanoma-pancreatic cancer syndrome (OMIM #606719). Increased risk of head and neck squamous cell carcinoma (HNSCC), particularly oral squamous cell carcinoma (OSCC) in those with germline *CDKN2A* mutations, has been described. However, screening for HNSCC is not a routine practice in patients with *CDKN2A* germline mutations and these mutations are not a conventional test for HNSCC patients without obvious risk factors.

**Case presentation:**

We describe a female with no smoking history who developed oral squamous cell carcinoma at age 39 and had a complex clinical course of recurrent multifocal squamous cell carcinoma (SCC) and carcinoma in situ of the oral cavity and oropharynx. Detailed family history demonstrated that her mother was diagnosed with OSCC and melanoma in her 40 s, and her maternal grandfather was diagnosed with metastatic melanoma in his 40 s. Genetic testing of the patient and her mother revealed *CDKN2A* c.301G>T mutation. She was referred to genetic counseling as well as to dermatology, gastroenterology, and neurology for cancer surveillance. She was treated with resections and has no evidence of disease 3 years after diagnosis.

**Conclusions:**

We report a family with a *CDKN2A* c.301 G>T mutation who also have significant history of OSCC, adding to the growing body of literature suggesting increased risk of HNSCC, particularly OSCC, in *CDKN2A* germline mutation carriers. It is important to consider *CDKN2A* mutation testing in familial HNSCC and young patients without obvious risk factors. Moreover, surveillance for HNSCC should be routine practice in those with a *CDKN2A* germline mutation.

**Graphical abstract:**

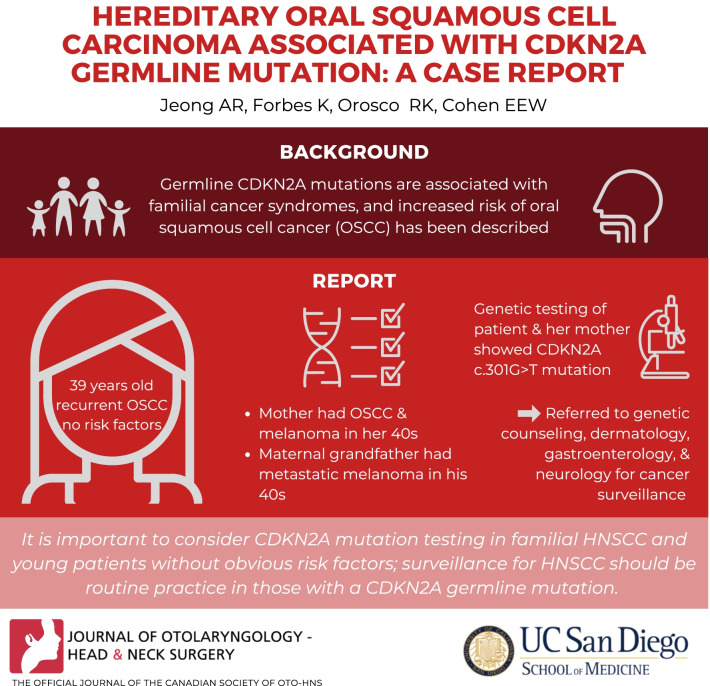

## Background

Cyclin dependent kinase inhibitor 2A (*CDKN2A*; OMIM 600160), located on 9p21.3, encodes proteins critical in cell cycle regulation. Through alternative reading frames, *CDKN2A* produces 2 major proteins: p16(INK4a), the cyclin dependent kinase (CDK) inhibitor, and p14(ARF), p53 stabilizer [1]. Germline, (inherited, in contrast to somatic or acquired), mutations of *CDKN2A* are the most common cause of inherited susceptibility to melanoma and is implicated in 25–40% of patients with familial atypical multiple mole melanoma (FAMMM; OMIM #155601) [2]. It is also associated with melanoma-pancreatic cancer syndrome (OMIM #606719), with an incidence of up to 60% depending on the type of mutation [2, 3]. Higher than expected frequencies of other types of cancers, such as head and neck squamous cell carcinoma (HNSCC), neural system tumors, GI cancer, breast cancer, and lung adenocarcinoma are reported in association with *CDKN2A* mutations, depending on the protein (either p14 or p16) affected [4]. However, there is no clear consensus on screening for cancers other than melanoma and pancreatic cancer. It is thus important to understand the association between *CDKN2A* mutation and development of non-melanoma and non-pancreatic cancer as patients should be appropriately counseled and monitored.

Here, we report a family with *CDKN2A* c.301G>T mutation who had multiple oral squamous cell cancers (OSCC) and melanoma. We review the growing body of literature of HNSCC in patients with *CDKN2A* mutations and propose that *CDKN2A* be a differential upon testing strategy in familial HNSCC, and that patients with *CDKN2A* mutation should be counseled regarding the risks of HNSCC.

### Case presentation

The patient is a 40-year-old female who was referred to our center for evaluation and treatment of recurrent Stage I (cT1N0M0) right tongue squamous cell carcinoma (SCC). The treatment history and pathology results are summarized in Table 1.

**Table 1:** Summary of treatment history and pathology results

Months from diagnosis	Treatment	Pathology
0	R partial glossectomy	Primary oral SCC
1	R partial glossectomy	Carcinoma in situ and severe dysplasia
11	R partial glossectomy	2 areas of carcinoma in situ
15	Pembrolizumab (off label)	
17	R partial glossectomy and oropharynx resection	high grade dysplasia and carcinoma in situ
19	Base of tongue resection	Mild dysplasia

She was diagnosed at age of 39, when she presented with pain and ulcer of her right tongue. Biopsy of the lesion demonstrated dyskeratosis but no malignancy. She experienced progressive pain and ulceration which led to right partial glossectomy. Pathology demonstrated SCC with positive anterior margin and dysplasia at the inferior margin. The tumor stained positively for p16 by immunohistochemistry. Subsequent positron emission tomography (PET)/computed tomography (CT) demonstrated 1.2 cm, PET-avid left thyroid nodule however no hypermetabolic lesions of the oral cavity or the cervical lymph nodes were demonstrated. Fine needle aspiration (FNA) of the thyroid nodule showed papillary thyroid carcinoma. She underwent total thyroidectomy, central neck dissection, and revision of her right partial glossectomy 5 weeks after her initial surgery. The pathology of her thyroid tissue showed Stage I (pT3bN1aM0) papillary thyroid carcinoma (PTC). Pathology of her R tongue resection showed severe dysplasia and carcinoma in situ. She received adjuvant radioactive iodine and is currently in remission from her PTC. Subsequently, she developed multiple recurrent oral squamous cell carcinoma in situ (and high grade dysplasia) despite resection. She was treated with pembrolizumab off-label at the outside clinic. When she developed a new high-grade dysplasia of the tongue, she was referred to our center for further management.

**Fig. 1:**
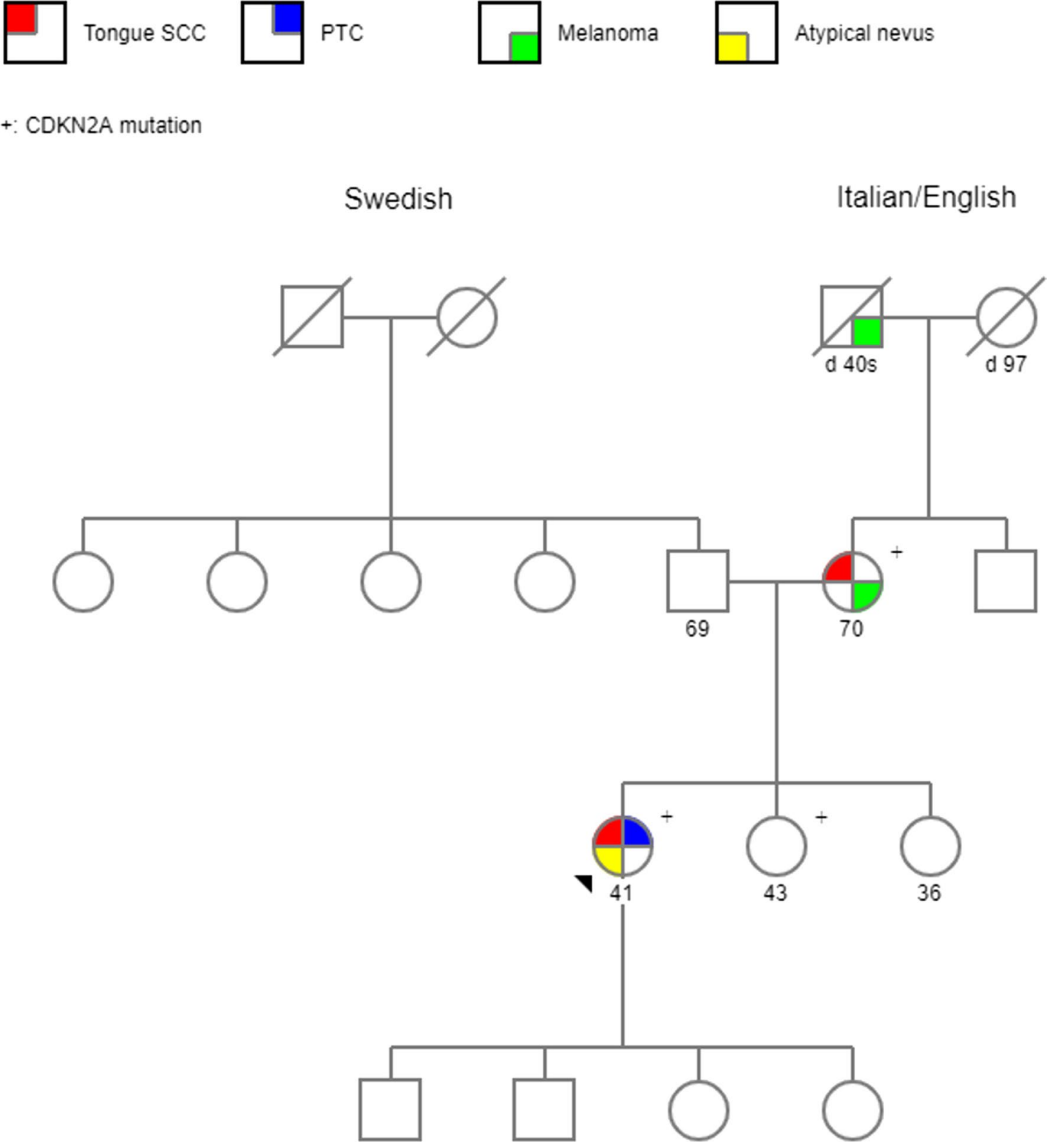
Pedigree. PTC, papillary thyroid carcinoma; SCC, squamous cell carcinoma

Her other medical history included post-thyroidectomy hypothyroidism and temporary hypoparathyroidism. As a result of immunotherapy, she developed type 1 diabetes mellitus. Her medications were calcitriol, levothyroxine, and insulin. Her mother, who is a life-long non-smoker, also has a history of early-stage melanoma at age 48, and tongue cancer at age 50 (Fig. 1). Her maternal grandfather was diagnosed with metastatic melanoma in his 40 s. There is no history of cancers on the paternal side. She denies any history of heavy alcohol use, smoking, or recreational drug use.

**Fig. 2:**
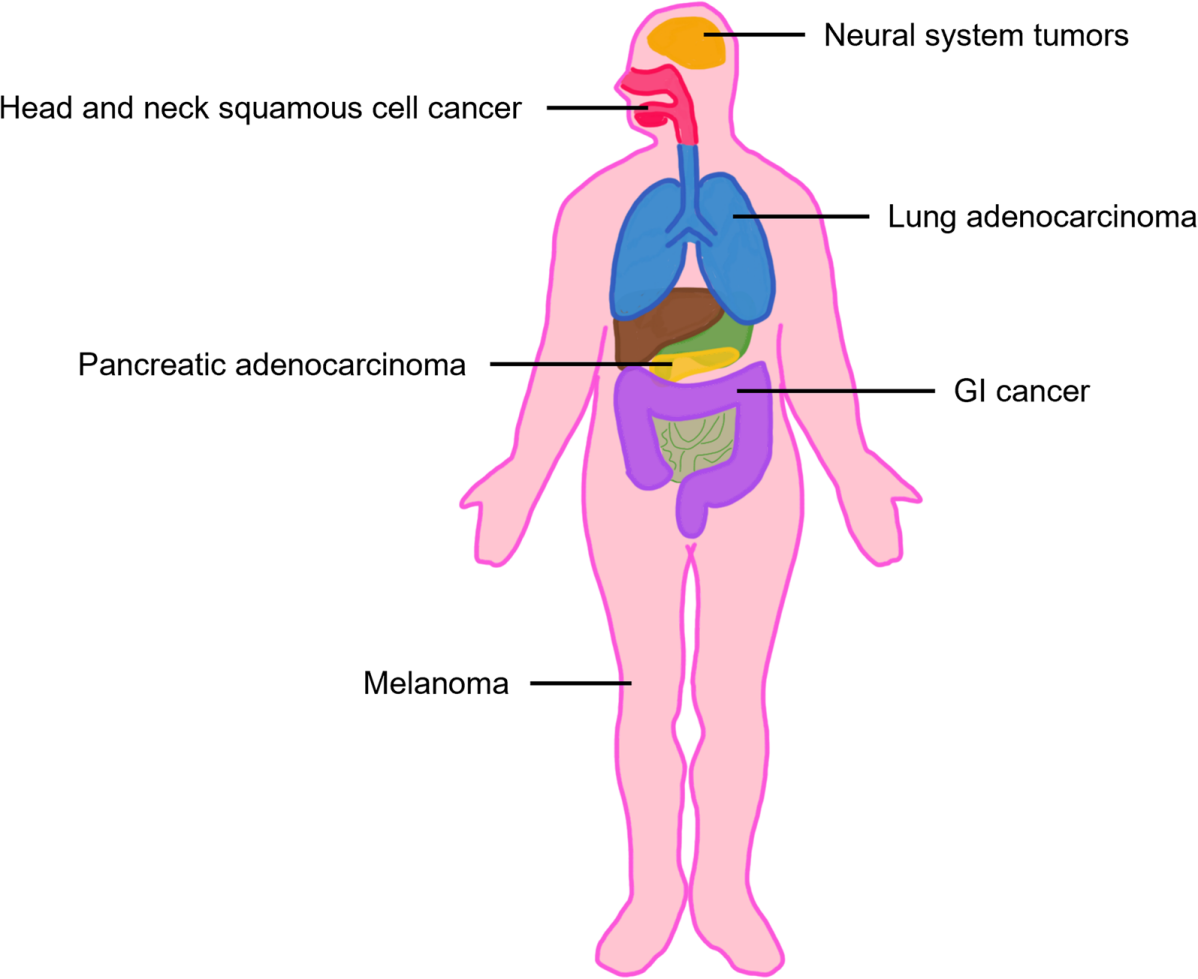
*CDK2NA* germline mutation associated malignancies

On evaluation, there was right tongue mucosal lesion that extended back to the oropharynx and a biopsy showed high grade dysplasia. She underwent a right partial glossectomy and right oropharynx resection. That specimen had carcinoma in situ, no invasive carcinoma, and two margins had mild dysplasia. Two months later, she was taken for a wide excision of the tongue base mucosa which was negative for dysplasia. Germline testing of 156 cancer related genes showed *CDKN2A* (c.301G>T) pathogenic mutation. Her mother and two siblings were also tested and her mother and sister were found to have the same germline mutation. Human Papilloma Virus (HPV) genotypes 16 and 18 DNA of the tongue biopsy specimen by polymerase chain reaction (PCR) were negative. She has now been without recurrence of SCC or carcinoma in situ for 18 months. She was referred to dermatology, gastroenterology, and neuro oncology for melanoma, pancreatic cancer, and brain tumor screening (Fig. 2).

## Discussion

We describe a case of a young female, life-long non-smoker, who presents with recurrent tongue SCC. Her case is unusual as she did not have significant risk factors for HNSCC such as tobacco use or alcohol use. Despite curative resection, she had multiple recurrent dysplasia and carcinoma in situ both at the resection margin and other parts of the tongue. Upon obtaining further history, her maternal family members had significant history of melanoma and HNSCC (Fig. 1). Patient’s mother, also a life-long non-smoker, was diagnosed with melanoma at age 48 and tongue cancer at age 50. The proband’s maternal grandfather had metastatic melanoma in his late 40 s. Genetic testing of both the proband and her mother revealed *CDKN2A* (c.301G>T) mutation.

*CDKN2A* (c.301G>T) mutation results in substitution of glycine with tryptophan at codon 101 (G101W) of the p16(INK4a) protein and replacement of arginine with leucine at codon 115 of the p14(ARF) protein. This variant is one of the most common types of *CDKN2A* mutations reported in Europe and North America [2]. It is particularly common in Italian families which this patient’s maternal ancestry traces back to. This mutant protein demonstrates significantly decreased binding of p16 to CDK4 and CDK6 [5].

The importance of p16 protein in the pathogenesis of various cancers have been well described. The p16 protein inhibits CDK4 and CDK6 and regulates cell cycle at the G1-S checkpoint [6]. Somatic mutations of *CDKN2A* are found in various sporadic forms of cancers. In HPV-negative HNSCC, somatic mutations of the p16/CDK-cyclin-D/retinoblastoma (Rb) pathway is one of the most common early mutations observed, reported in approximately 20% of cases [7, 8]. In HPV positive HNSCC, the oncogene E7 inhibits Rb protein which leads to epigenetic de-silencing of *CDKN2A* gene, and subsequent increased expression of p16INK4a [9].

Given its important function in tumorigenesis, loss of the wild type *CDKN2A* allele in those with a germline mutation is likely a key event in development of HNSCC. Patient who is a carrier of abnormal *CDKN2A* gene is asymptomatic until the normal *CDKN2A* gene acquires a loss-of-function mutation (also termed loss of heterozygosity). Loss of heterozygosity is reported in all HNSCC cases with a germline *CDKN2A* mutation, in those who were tested [10–15]. Thus, patients who acquire mutation of the wild type *CDKN2A* gene by environmental or secondary factors may be at increased risk of developing HNSCC. In our patient, not enough tissue was available for next generation sequencing (NGS), thus loss of heterozygosity was not able to be demonstrated.

Interestingly, our patient demonstrated p16 positivity by immunohistochemistry (IHC). None of the reported cases of HNSCC in those with *CDKN2A* germline mutation demonstrated p16 positivity: either the patients had negative p16 expression by IHC [12, 13]. or patients whose tumor was p16 negative were selected for study [10]. This finding may be expected as *CDKN2A* codes for the p16 protein, and mutation in *CDKN2A* could result in loss of expression. Furthermore, p16 expression by IHC is a screening marker for HPV related HNSCC. Notably, G101W mutant p16 were shown to localize to nucleus in vitro and in melanocytes [16, 17]. In melanoma, mutant p16 expression decreased with more advanced stages of disease [16]. Therefore, it is not surprising that p16 was detected by IHC in this early stage tongue cancer. HPV genotype 16 and 18 DNA were negative ruling out an HPV driven process. It is important to consider *CDKN2A* mutation in familial HNSCC even if p16 is positive by IHC and should be confirmed by PCR testing.

*CDKN2A* germline mutations are implicated in FAMMM. Although there is clear association with increased risk of cutaneous melanoma, the risk of mucosal melanoma is less well defined. It is also associated with development of hereditary pancreatic cancer syndromes [4, 18], and surveillance detected pancreatic cancer was more likely to be resectable and patients had higher overall survival rate at 5 years compared to historical control [19]. Due to the increased risk of melanoma and pancreatic cancer, current guidelines suggest total body skin exams, including oral mucosa, genital area, and nails at 3–6 month intervals starting late adolescence [20] and annual screening for pancreatic cancer starting at age 40 with magnetic resonance imaging (MRI) and endoscopic ultrasonography (EUS) [21]. Increased risk of neural systems tumor (melanoma-astrocytoma syndrome; OMIM #155755) is also reported [18, 22], although there is no formal screening guideline for astrocytoma. In contrast, *CDKN2A* germline mutation as a cause of familial HNSCC is not widely accepted, and patients are not screened for HNSCC. There is a growing body of evidence that families with germline *CDKN2A* mutations are at increased risk of HNSCC, particularly oral SCC (Table 2) [10–15, 23–27].

**Table 2:** Summary of reported cases of HNSCC with confirmed underlying *CDKN2A* germline mutation

References	Patient ID^a^	Mutation	LOH	Type of HNSCC	p16 IHC
Whelan et al.[26]	IV-1	G101W	N/A	OSCC (tongue)	N/A
Sun et al.[25]	I-1	c.G159C (M53I)	N/A	OSCC	N/A
	III-1	c.G159C (M53I)	N/A	OSCC	N/A
Yarbrough et al. [14]	Proband’s niece	P16(Δ96-99)	Yes	HNSCC	N/A
Della Torre et al. [24]	III-17 (2587)	P48T	N/A	OSCC	N/A
Yu et al. [15]	II-A	c.G260C (R87P)	Yes	OSCC (tongue)	N/A
	II-D	c.G260C (R87P)	Yes	HNSCC	N/A
	II-I	c.G260C (R87P)	Yes	HNSCC	N/A
Schneider-Stock et al. [12]	Proband	P16-Leiden	Yes	OSCC	Neg
Oldenburg et al. [11]	36 (EMC 13769)	p16-Leiden	N/A	OSCC (tongue)	N/A
	48 (EMC 13769)	p16-Leiden	Yes	OSCC	Neg
Vinarsky et al. [13]	IIa	c.G302T (G101W)	Yes	OSCC (tongue)	N/A
Cabanillas et al. [23]	III-15	c.106delG	N/A	OSCC (hard palate)	N/A
	III-16	c.106delG	N/A	Hypopharyngeal SCC	N/A
Fostira et al. [10]	OT-10	c.G71C	Yes	OSCC	Neg
	OT-14	c.G71C	Yes	OSCC	Neg
Chan et al. [27]	Proband	Deletion CDKN2A-CDKN2B	Yes	Laryngeal SCC	Neg

Those patients who have confirmed mutation by sequencing or obligate carriers are included

^a^As published in the original article

HNSCC, head and neck squamous cell carcinoma; IHC, immunohistochemistry; LOH, loss of heterozygosity; N/A, not available; Neg, negative; OSCC, oral squamous cell carcinoma; SCC, squamous cell carcinoma

In a population-based study of Icelandic patients with melanoma and controls, Goldstein and colleagues [28] identified an Icelandic founder mutation G89D with allelic frequency of 0.7% among melanoma patients. The Icelandic patients with melanoma who had G89D mutation also had familial relative risk for HNSCC of 4.84 (*p* = 0.016).

*CDKN2A* mutation was also demonstrated in a prospective group of OSCC patients. Fostira and colleagues [10] analyzed patients who were less than 50 years old and with a history of HPV negative OSCC for any predisposing germline mutations. Among 30 patients who were prospectively enrolled, 4 patients were found to have potential pathogenic germline mutations. Two of the 4 unrelated patients had *CDKN2A* c.71G>C mutation and both exhibited LOH on the tumor sample testing. Both had significant smoking history prior to diagnosis.

In addition to the increased risk of melanoma, pancreatic cancer, and neural systems tumor, various cancers have been reported in patients with germline *CDKN2A* mutation. These include breast adenocarcinoma [4, 29–34], lung adenocarcinoma [4, 33, 35], sarcoma [35–37], and neurofibroma [22, 31, 34, 38–40], and the available literature is summarized in Table 3.

**Table 3:** Reports of selected neoplasms in association with germline CDKN2A mutation

Type of cancer	References	Comments
Breast adenocarcinoma	[29]	Standardized morbidity rate of 3.8 (*P* = 0.0014)
	[30]	Case report
	[31]	One pedigree described. Breast cancer in 5 members
	[32]	2 cases series of breast cancer
	[34]	One pedigree described. Breast cancer in 1 member
	[4]	Large registry study. Prevalence rate was 3.044 compared to *CDKN2A* wild type (*P* < 0.001)
	[33]	One pedigree described. Breast cancer in proband
Lung cancer	[33]	One pedigree described. Lung cancer in 3 members
	[4]	Large registry study. Prevalence rate was 2.19 compared to *CDKN2A* wild type (*P* < 0.018)
	[35]	Eight pedigrees described. Lung cancer in 6 members
Sarcoma	[37]	Eight cases of sarcoma identified in melanoma -prone French families with *CDKN2A* germline mutation
	[35]	Eight pedigrees described. Sarcoma in 5 members
	[36]	744 pediatric patients were screened for germline mutations: 1 patient with osteosarcoma found to have *CDKN2A* germline mutation
Neurofibroma	[39]	Case report
	[22]	In one pedigree, 7 members had neural system tumors including astrocytoma, neurofibroma, schwanomma, and meningioma
	[38]	One pedigree described. Neurofibroma in 4 members
	[31]	One pedigree described. Neurofibroma in 3 members
	[34]	One pedigree described. Neurofibroma in proband
	[40]	One pedigree described. Neurofibroma in 3 members

Although limited by the small number of cases, this report adds to the growing body of evidence that *CDKN2A* germline mutations predispose to the development of HNSCC. The proteins encoded by *CDKN2A* play critical roles in pathogenesis of HNSCC and LOH is likely a key early event. Our case is unique due to p16 positivity by IHC in the tumor. This finding does not necessarily represent HPV infection and when suspicious, should be confirmed by HPV genotyping by PCR. Furthermore, p16 positivity by IHC should not preclude familial HNSCC syndrome. Future studies should prospectively investigate the risk of HNSCC in those with a *CDKN2A* germline mutation. It is important to consider *CDKN2A* mutation testing in familial HNSCC, even when p16 testing by IHC is positive, and patients with a *CDKN2A* mutation should be counseled regarding the potential increased risk of HNSCC. In addition, routine screening of HNSCC should be considered, patients should avoid known risk factors such as smoking and alcohol, and all patients should receive HPV vaccination.

## Conclusions

We report a young female with no significant risk factors who developed tongue SCC. Family history revealed mother with oral SCC. Both the patient and her mother were found to have *CDKN2A* c.301G>T germline mutation. *CDKN2A* germline mutations may increase the risk of development of HNSCC and patients should be appropriately counseled and monitored for HNSCC.

### Patient perspectives

“No one wants to hear that news that they have cancer. I had a pretty hard battle with multiple recurrences and multiple surgeries. When I came to UCSD I was on my third recurrence, feeling pretty discouraged. I have received excellent care from my current team of doctors. Adding genetic testing and counseling to my care provided some much-needed answers not only for me, but my family as well. I'm on my longest stretch of being disease free and am feeling well and thankful. Hopefully finding this link between the mutation and OSCC will help others in the future.”

## Data Availability

Not applicable.

## Abbreviations

CDK: Cyclin dependent kinase
CDKN2A: Cyclin dependent kinase inhibitor 2A
CT: Computed tomography
FAMMM: Familial atypical multiple mole melanoma
FNA: Fine needle aspiration
HNSCC: Head and neck squamous cell carcinoma
HPV: Human papilloma virus
IHC: Immunohistochemistry
NGS: Next generation sequencing
OSCC: Oral squamous cell cancers
PCR: Polymerase chain reaction
PET: Positron emission tomography
PTC: Papillary thyroid carcinoma
Rb: Retinoblastoma
SCC: Squamous cell carcinoma
